# Establishing Hematopoietic Stem Cell Transplant Unit in Resource Limited Setting: A Critical Analysis of Indian Council of Medical Research 2017 Guidelines

**DOI:** 10.1155/2018/1292307

**Published:** 2018-08-08

**Authors:** Kunal Das, Tanvi Khanna, Nitika Agrawal

**Affiliations:** ^1^Cancer Research Institute, Swami Rama Himalayan University, Dehradun, India; ^2^Himalayan Institute of Medical Sciences, Swami Rama Himalayan University, Dehradun, India

## Abstract

The scope and application of hematopoietic stem cell transplantation are increasing. With advancement in science and close cooperation of health centers, HSCT units are coming up in new developing and underdeveloped countries. India hosts many HSCT units and often provides financially viable option for HSCT to foreign patients as well. Recently Indian Council of Medical Research (ICMR) issued a guideline about HSCT unit in India. This review article discusses establishment of new HSCT unit in resource limited setting. Subsequent implication of ICMR guideline has been done.

## 1. Introduction

Hematopoietic stem cell transplant (HSCT) is often the only curative option in many hematological malignant and nonmalignant conditions. The indication of HSCT is ever increasing and world has witnessed plethora of HSCT being conducted at present in comparison to past [1]. Stem cell transplant is a rapidly increasing health facility in India. India caters patients in need of HSCT from abroad as well since it is much affordable here [2]. HSCT activity parallels the economic growth of countries and HSCT activities have been noted to be absent in countries with low GDP [680 US dollar] and low population density [3]. Unfortunately the disease does not follow similar pattern and huge unmet need in developing/underdeveloped countries is persistent. Establishing a HSCT unit in resource limited setting remains a challenge. Social and economic distribution are quite variable in India. Even increased number of HSCT units in India is falling short for these needs. Indian Council of Medical Research (ICMR) has issued a guideline/directives for minimum standers required to run a HSCT unit [4]. This article reviews the need of unit, real issues, while establishing and running it and ICMR guideline is pertaining it.

## 2. Assessment of Need

To establish a HSCT unit, an assessment of need should be done. A proper requirement is not based on presence or absence of individual disease, but on burden of disease in society. A disease like sickle cell anemia or thalassemia is endemic in certain geographical area and operating a HSCT unit is expected to be cost effective and successful in long run there. Malignancies are having uneven geographical distribution as well and hematological malignancies might require HSCT at different point of treatment or relapse [5–7]. A density of population and incidence of such ailment can be a guide for HSCT programme establishment. However lack of reliable and accurate data and absence of guideline for such exercise is hindrance in assessing need in developing/underdeveloped countries [8]. Nigeria has endemic sickle cell disease affecting almost 2-3% of total population. The indirect and direct cost of care are huge. Thus establishing a HSCT unit for allogeneic type of HSCT was a cost effective decision [9]. For low population countries like Maldives and Mauritius the density of HSCT candidates is too less to run a programme perennially. State sponsored shifting to other countries for HSCT is a cost effective method. However, by this a lack of science and knowledge will continue [10].

India is a large country with quite variable socioeconomic strata. Majority of rural population are still finding it hard to meet health need, while on the other hand, it hosts many foreign national a medical tourism [11]. ICMR guideline is silent on criteria of opening a HSCT center. No mention of state policy, population need assessment, or population density has been done [4]. A functional oncology-hematology service is required for need assessment and proper recruitment. It is recommended to run such health center for 3-5 years before starting CT services. This time period will also help in arranging resources and getting exposure for supportive care.

## 3. State versus Private

For a HSCT facility to come up, apart from huge cost, health worker training, location, equipment, blood bank, and laboratory advancement are needed [12]. State with available budget can establish a HSCT center easily as recovery of cost is not a target. However private parties investing huge sums in HSCT is expected to charge at higher pace for recovery of cost. In countries/state where GDP is low, state should take initiative to establish HSCT facility. A running cost should be anticipated and charges should be framed accordingly. Bangladesh sets an example of running good health services including HSCT program despite low GDP. State sponsorship as well as twining with established center helped in achieving this target [13]. Unfortunately, in India, majority of SCT centers are run by private finance, although state subsidies in land, electricity, and other taxes are there. State owned SCT units are few and cater very limited number of cases.

## 4. Infrastructure

HSCT unit should be a segregated but well-connected area of hospital. While design and HEPA filtration can be done by seeking services of professionals, a help from already established center will be beneficial [14]. The area of HSCT should be away from dampness, infection wards, and construction site. Access to support services like radiology, laboratory, and blood bank should be kept in mind while designing the facility. Preferably all these facilities should be in same building. The maintenance of HSCT unit will be required periodically. Special attention should be paid for designing and servicing of air conditioner, sluice room, drains, and toilets. The maintenance should be done without actual entry into unit. Service ducts access should be available from outside. Design of unit should be done keeping long-term sustainability and target in mind [8]. At present, there is no guideline which defines minimum infrastructure needed to start the unit. However ICMR guideline 2017 mentions positive pressure airflow system, a requisite for the unit. A unit must have minimum of 2 rooms as per guideline [4]. Guideline states that these rooms can be a part of immune-compromised patient care unit or leukemia care unit.

## 5. Support Services

HSCT facility is not an isolated entity. Its functioning depends on multiple support system which includes a separate out-patient clinic and recruitment area, patient screening services with cardiology, pulmonology, dental, and psychiatry. Blood bank facility should be available and with proper license for apheresis, blood components, and stem cell collection and storage. Availability of blood components should be in abundance and regular camp/donation must be in place. Blood irradiation facility is also essential. Laboratory requirement of HSCT is different. Some tests are routine and require in-house facility like complete hemogram, flow cytometry capable of doing CD34 counts, liver and kidney function tests, electrolyte, and sepsis screening. Facilities like viral assay, HLA typing, donor specific antibody screen, chimerism, fluorescent in situ hybridization, and drug levels can be outsourced to central laboratory. Establishing advanced and molecular lab will be a costly affair; central pooling of samples is possible and cost effective way [15]. However an audit for the need and cost effectiveness for all outsourced facility should be done regularly. Radiotherapy facility is required for some of conditioning regimen but not all. For initial HSCT, a conditioning regimen without radiation can be selected [16, 17].

ICMR guideline enlists blood bank with component availability as a minimum requirement. A 24-hour blood bank, microbiology, pathology, and biochemistry support is essential. There is no list of minimum investigation suggested, leaving a scope of outsourcing advance tests. Guideline suggests in-house HLA typing or enlisting a registered HLA lab as outsource facility. Similarly guideline which enlists dialysis facility and intensive care facility should be present in house to deal with complications [4]. Use of word “should be” makes it suggest rather than mandate such vital support services. For state owned units, swapping of staff for limited period may help in developing this capacity. In India, on request, curriculum based observership at an established SCT unit is a usual practice to gain experience and confidence.

## 6. Person Training

Sustained availability of trained personnel is one of the main hurdles in advanced care units. It includes clinician, staff nurses, lab technicians, blood bank staff, social worker, and psychologist. Whole hospital needs to gear up for such advance facility. A training of health persons directly involved in patient care is feasible with hiring a trained one, fellowship, observership, or twining. Twining or pairing with established HSCT center gives a better and cost effective opportunity for training, orientation, and guidance as well [14, 18]. Bangladesh stem cell transplant programme is an elaborated example of twining and collaboration. Ministry of health and family welfare of Bangladesh collaborated with Massachusetts General Hospital for starting stem cell transplant facility. The collaboration included exchange and training of faculties and staffs, joint capacity building exercise for support staff training, and joint monitoring of activities. Dhaka stem cell programme collaboration was a 150 weeks of training before performing first HSCT [13]. They incorporated suggestions from developing countries regarding low budget programme and air and water contamination issues [19]. Similar twining is desired to achieve a safe and comfortable expertise of starting stem cell transplant activity.

Twining with an established center has shown monitored and tailored growth of HSCT activity in Rajasthan, India, as well. Cure2children, a United States based nonprofit organization, helped in establishing an allogeneic HSCT unit in Rajasthan. Six volunteer doctors and four specialized nurses trained new team at this place to start the program. Now this is an independent HSCT program [20].

ICMR guideline delineates the qualification and experience of the in-charge of HSCT unit in detail. It includes training abroad but limits the scope to America, England and Australia trained person only [4]. A requisite is of special training in recognized Department of Hematology and HSCT Unit has been drafted. Guideline is not clear if a doctor trained from Singapore/Italy wants to practice, what additional training is required. Probably a list of reputed HSCT/hematology units across the globe will do more justice in recognizing training than a country specific. There is no mention about staff qualification or training module. Guideline did not mention twinning/pairing.

## 7. Patient Selection

Selection of case not only is important for clinician and hospital morale but has a bearing on society also. Every HSCT unit wishes for recovery of all cases but relapse, failure, and death are clinical outcome in some of cases. A wise selection of cases at onset will boost the morale of staff and put public faith in HSCT unit. Although all HSCT requires high level of clinical acumen, an autologous HSCT of multiple myeloma is relatively less complicated than an allogeneic HSCT for adult with thalassemia major. Similarly among allogeneic HSCT, nonmalignant condition like aplastic anemia is more likely to produce a cure rather than refractory leukemia [21]. There is always a reluctance in society to be the first case; a proper counselling and good selection is helpful to start the unit.

ICMR guideline states that center has to acquire experience in HSCT and minimum 20 allogeneic HSCT must be done in that center before proceeding to haploidentical, matched unrelated transplant or cord blood transplant. It does not give any weightage to the transplant physician expertise for that. A team which has done a large number of HSCT, if it starts another center, as per guideline, must start with low risk HSCT. Guideline also defines a minimum of 10 HSCT [for autologous and allogeneic each] per year for a running unit. But if it is not happening due to any reason, it is not clear about remedial measure. Guideline also suggests not to do transplant for rare conditions but has not elaborated the experience required to proceed for such HSCT. Patients with such uncommon diseases should not be deprived of cure chances. A collaboration with established SCT unit can help in diverting high risk patient and recruiting low mortality cases for initial SCT.

## 8. Finance

HSCT is an expansive modality requiring lots of medication, blood products, supportive care apart from infrastructure, and human cost. Direct cost, although seems less, when added with indirect costing makes it unaffordable for majority. A charge should be framed based on real expanses as well as paying capacity of society. State/insurance sponsorship is desired. A long-term cot recovery plan should be considered while framing charges. The cost to patient is quite variable across the globe. While costing in India and Mexico is US$ 17,194 and 12,500, cost of HSCT in Europe and America is comparatively very high, US$ 120,000 [22–24]. As the GDP per capital of these countries are different and health care support is also not matching, it is not prudent to consider these cost figures as direct difference due to geography. To curtail the cost of HSCT, pooling of resources like central laboratory and molecular laboratory facility, central/shared blood irradiation facility can be done. Step down isolation ward can reduce the burden on the unit and reduce the cost. Patient data and clinical records should be pooled to increase the experience and knowledge. Fund raising and awareness for it are essential components for arranging the cost [22]. State sponsorship and donation/help from nongovernment organization must be active for HSCT programme. Our SCT programme got full financial support from government for children; adult cases are raising funds from government and nongovernment organizations. However some centers pool the fund using fixed package for SCT. Social media and local newspaper also help in fund raising.

## 9. Conclusion

HSCT unit is an advanced setup and its scope is widening. ICMR has recently released minimum stander for HSCT unit which defines clearly about unit infrastructure, support, and personnel. But this guideline is a crude instructive framework and majority of fields are left open for variation. A detailed module for infrastructure, personnel, and support staff is required. Use of word “should be” and “must be” needs further clarification. Probably, a revised guideline in subsequent years will address these issues. Authors strongly recommend twining for a new SCT unit.
